# Supplementary material for: Adding PD-1/PD-L1 Inhibitors to Chemotherapy for the First-Line Treatment of Extensive Stage Small Cell Lung Cancer (SCLC): A Meta-Analysis of Randomized Trials
Source: Cancers (Basel). 2020 Sep 16;12(9):2645. doi: 10.3390/cancers12092645 (PMC7565587; doi:10.3390/cancers12092645)
Supplement: Supplementary file 1 [file cancers-12-02645-s001.pdf]

cancers

Supplementary Material

Adding PD-1/PD-L1 Inhibitors to Chemotherapy for the First-Line Treatment of Extensive Stage Small Cell Lung Cancer (SCLC): A Meta-Analysis of Randomized Trials

Francesco Facchinetti, Massimo Di Maio, Marcello Tiseo

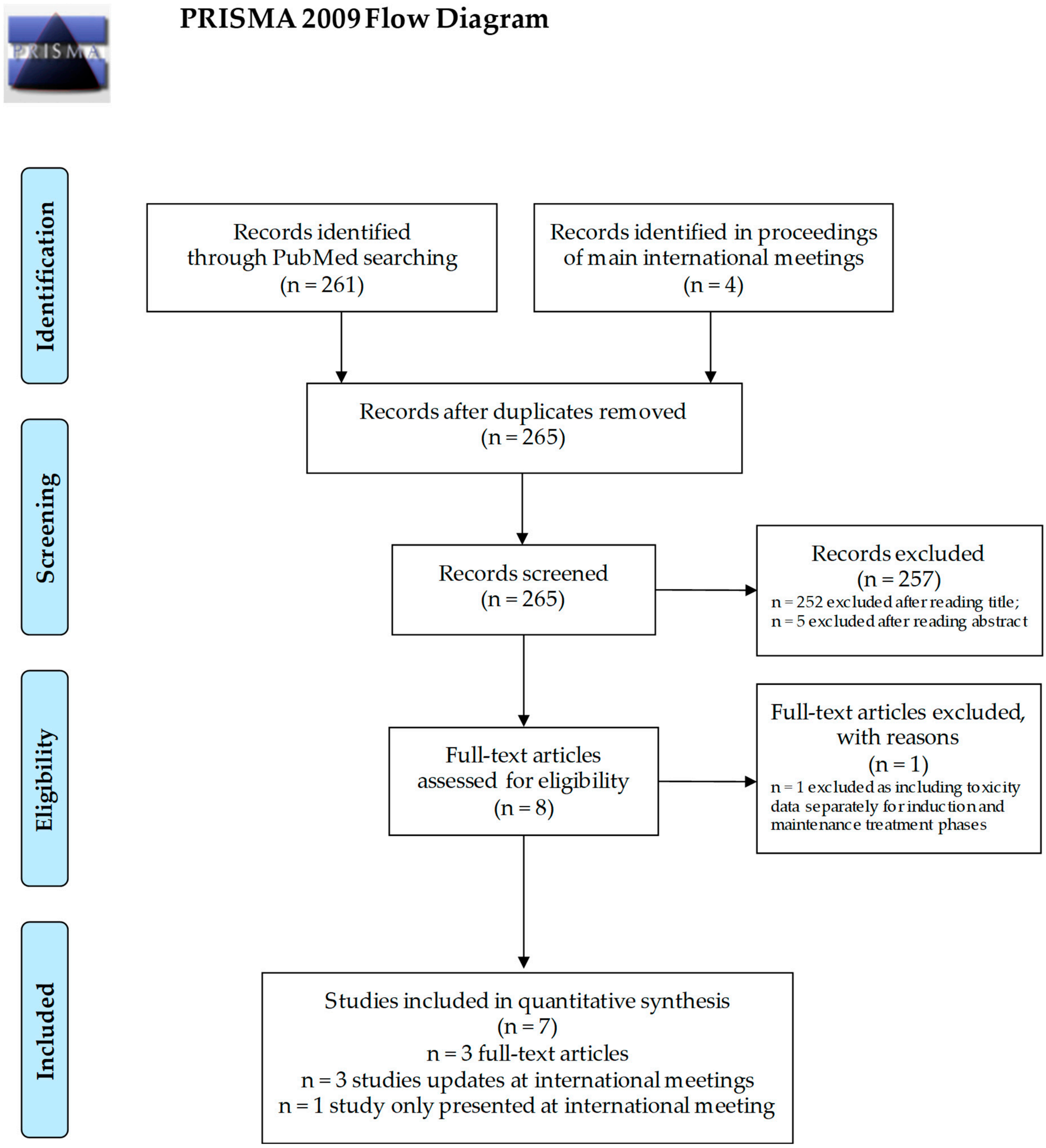

Figure S1. PRISMA flow diagram depicting the systematic review process leading to the identification of studies included in the meta-analysis [1].

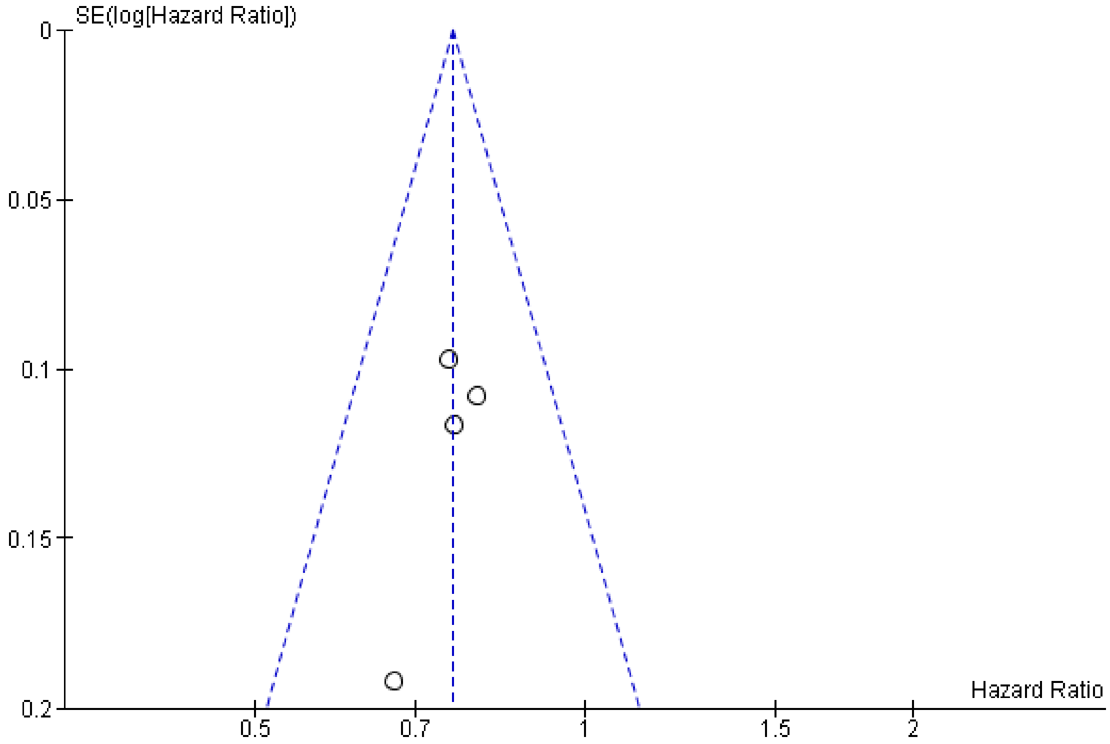

Figure S2. Funnel plot for overall survival, showing the distribution of effect estimates (hazard ratio) against standard error of log(hazard ratio). SE: standard error.

Study or Subgroup	log[Hazard Ratio]	SE	Weight	Hazard Ratio IV, Fixed, 95% CI
IMpower 133	-0.2758	0.1165	24.6%	0.76 [0.60, 0.95]
CASPIAN	-0.2877	0.1317	19.3%	0.75 [0.58, 0.97]
CASPIAN durvalumab + tremelimumab	-0.1972	0.1344	18.5%	0.82 [0.63, 1.07]
KEYNOTE-604	-0.2282	0.1081	28.6%	0.80 [0.64, 0.98]
ECOG-ACRIN EA5161	-0.4005	0.1919	9.1%	0.67 [0.46, 0.98]
**Total (95% CI)**			**100.0%**	**0.77 [0.69, 0.86]**

Heterogeneity: Chi² = 0.90, df = 4 (P = 0.92); I² = 0%
Test for overall effect: Z = 4.52 (P < 0.00001)

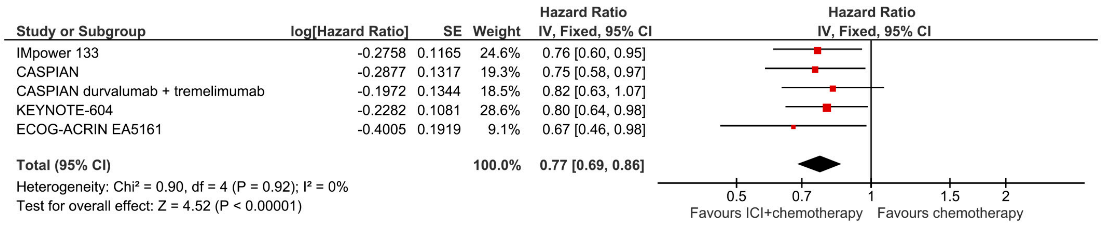

Figure S3. Overall survival plots considering CASPIAN arm with chemotherapy + durvalumab + tremelimumab. IV: Inverse variance; 95% CI: 95% confidence interval.

A. All adverse events.

	ICI + chemotherapy		Chemotherapy			Odds Ratio	
Study or Subgroup	Events	Total	Events	Total	Weight	M-H, Fixed, 95% CI	Year
IMpower 133	198	198	189	196	8.2%	15.71 [0.89, 277.02]	2018
CASPIAN	260	265	258	266	83.3%	1.61 [0.52, 4.99]	2019
KEYNOTE-604	223	223	222	223	8.5%	3.01 [0.12, 74.37]	2020
**Total (95% CI)**		**686**		**685**	**100.0%**	**2.89 [1.13, 7.38]**	
Total events	681		669				

Heterogeneity: Chi² = 2.36, df = 2 (P = 0.31); I² = 15%
Test for overall effect: Z = 2.22 (P = 0.03)

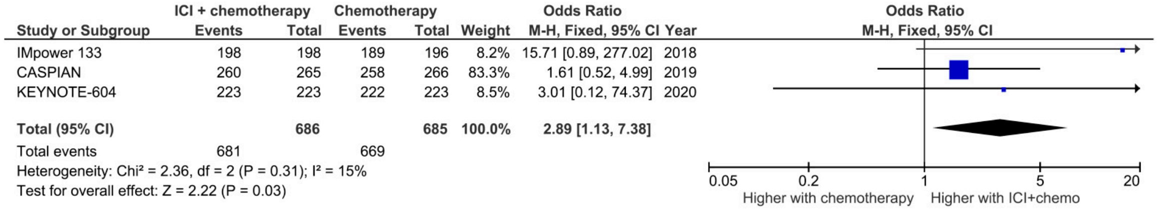

B. Grade 3-4 adverse events

Study or Subgroup	ICI + chemotherapy Events	Total	Chemotherapy Events	Total	Weight	Odds Ratio M-H, Fixed, 95% CI	Year
IMpower 133	134	198	124	196	28.3%	1.22 [0.80, 1.84]	2018
CASPIAN	165	265	167	266	44.3%	0.98 [0.69, 1.39]	2019
KEYNOTE-604	171	223	167	223	27.4%	1.10 [0.71, 1.70]	2020
**Total (95% CI)**		**686**		**685**	**100.0%**	**1.08 [0.86, 1.36]**	
Total events	470		458				

Heterogeneity: Chi² = 0.63, df = 2 (P = 0.73); I² = 0%
Test for overall effect: Z = 0.66 (P = 0.51)

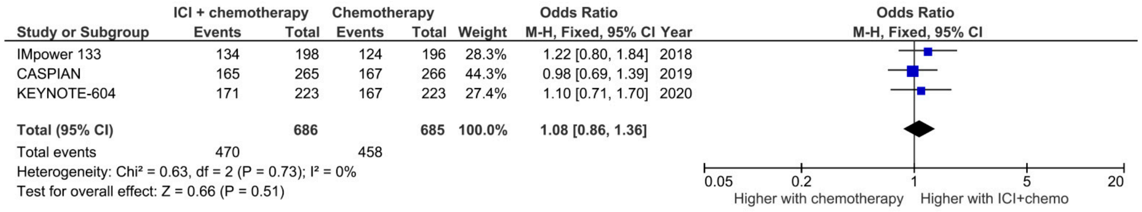

C. Immune-related adverse events

Study or Subgroup	ICI + chemotherapy Events	Total	Chemotherapy Events	Total	Weight	Odds Ratio M-H, Fixed, 95% CI	Year
IMpower 133	82	198	48	196	55.2%	2.18 [1.42, 3.35]	2018
CASPIAN	53	265	7	266	10.9%	9.25 [4.12, 20.77]	2019
KEYNOTE-604	55	223	23	223	33.9%	2.85 [1.68, 4.83]	2020
**Total (95% CI)**		**686**		**685**	**100.0%**	**3.18 [2.35, 4.29]**	
Total events	190		78				

Heterogeneity: Chi² = 9.81, df = 2 (P = 0.007); I² = 80%
Test for overall effect: Z = 7.54 (P < 0.00001)

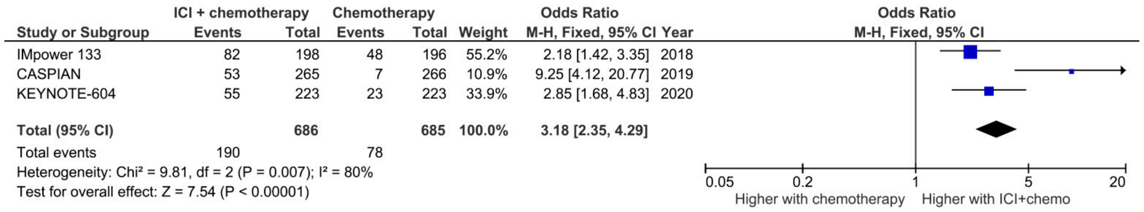

D. Adverse events leading to withdrawal of any treatment

Study or Subgroup	ICI + chemotherapy Events	Total	Chemotherapy Events	Total	Weight	Odds Ratio M-H, Fixed, 95% CI	Year
IMpower 133	24	198	6	196	13.4%	4.37 [1.74, 10.94]	2018
CASPIAN	27	265	25	266	56.5%	1.09 [0.62, 1.94]	2019
KEYNOTE-604	33	223	14	223	30.1%	2.59 [1.35, 4.99]	2020
**Total (95% CI)**		**686**		**685**	**100.0%**	**1.98 [1.36, 2.89]**	
Total events	84		45				

Heterogeneity: Chi² = 7.63, df = 2 (P = 0.02); I² = 74%
Test for overall effect: Z = 3.54 (P = 0.0004)

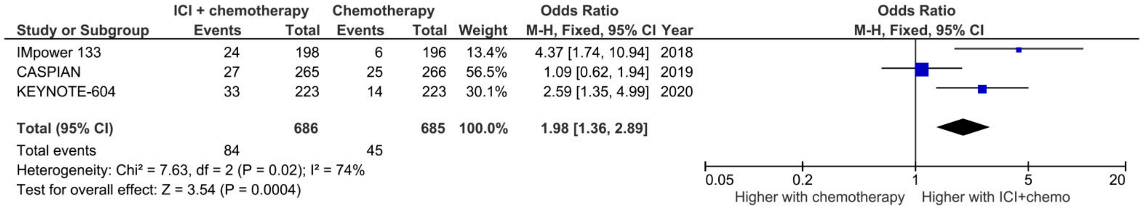

E. Grade 5 treatment-related adverse events

Study or Subgroup	ICI + chemotherapy Events	Total	Chemotherapy Events	Total	Weight	Odds Ratio M-H, Fixed, 95% CI	Year
IMpower 133	3	198	3	196	25.2%	0.99 [0.20, 4.96]	2018
CASPIAN	6	265	2	266	16.6%	3.06 [0.61, 15.29]	2019
KEYNOTE-604	6	223	6	223	49.6%	1.00 [0.32, 3.15]	2020
ECOG-ACRIN EA5161	1	75	1	70	8.7%	0.93 [0.06, 15.20]	2020
**Total (95% CI)**		**761**		**755**	**100.0%**	**1.33 [0.63, 2.84]**	
Total events	16		12				

Heterogeneity: Chi² = 1.46, df = 3 (P = 0.69); I² = 0%
Test for overall effect: Z = 0.74 (P = 0.46)

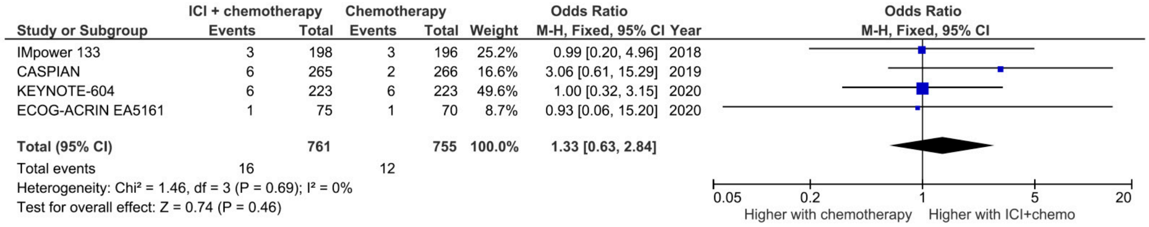

F. Dermatitis / rash, all grades

Study or Subgroup	ICI + chemotherapy Events	Total	Chemotherapy Events	Total	Weight	Odds Ratio M-H, Fixed, 95% CI	Year
IMpower 133	40	198	21	196	56.0%	2.11 [1.19, 3.73]	2018
CASPIAN	4	265	2	266	6.5%	2.02 [0.37, 11.14]	2019
KEYNOTE-604	30	223	13	223	37.4%	2.51 [1.27, 4.95]	2020
**Total (95% CI)**		**686**		**685**	**100.0%**	**2.25 [1.48, 3.44]**	
Total events	74		36				

Heterogeneity: Chi² = 0.16, df = 2 (P = 0.92); I² = 0%
Test for overall effect: Z = 3.77 (P = 0.0002)

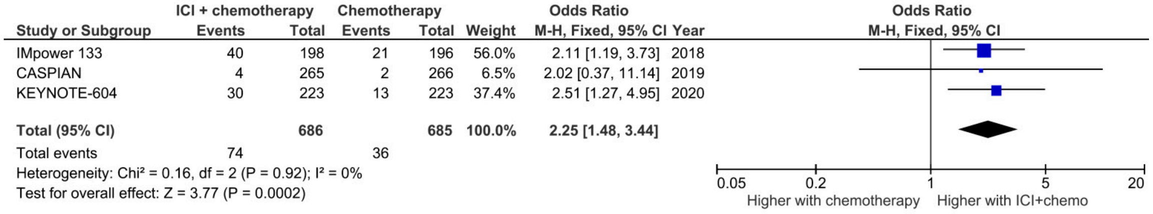

H. Dermatitis / rash, grade 3-4

Study or Subgroup	ICI + chemotherapy Events	Total	Chemotherapy Events	Total	Weight	Odds Ratio M-H, Fixed, 95% CI	Year
IMpower 133	4	198	0	196	49.9%	9.09 [0.49, 170.02]	2018
CASPIAN	0	265	0	266		Not estimable	2019
KEYNOTE-604	3	223	0	223	50.1%	7.10 [0.36, 138.16]	2020
**Total (95% CI)**		**686**		**685**	**100.0%**	**8.09 [1.01, 64.99]**	
Total events	7		0				

Heterogeneity: Chi² = 0.01, df = 1 (P = 0.91); I² = 0%
Test for overall effect: Z = 1.97 (P = 0.05)

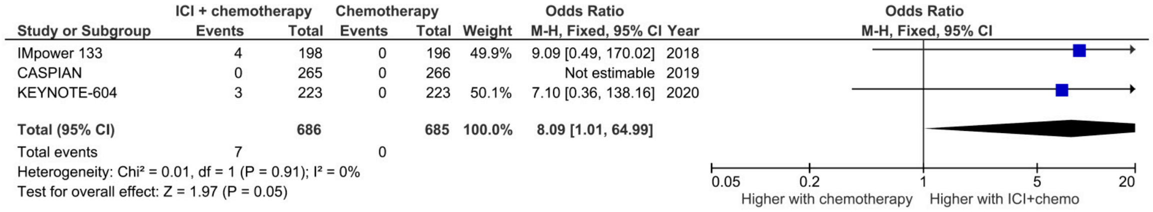

J. Hepatitis, all grades

Study or Subgroup	ICI + chemotherapy Events	ICI + chemotherapy Total	Chemotherapy Events	Chemotherapy Total	Weight	Odds Ratio M-H, Fixed, 95% CI	Year
IMpower 133	4	198	0	196	49.9%	9.09 [0.49, 170.02]	2018
CASPIAN	0	265	0	266		Not estimable	2019
KEYNOTE-604	3	223	0	223	50.1%	7.10 [0.36, 138.16]	2020
**Total (95% CI)**		**686**		**685**	**100.0%**	**8.09 [1.01, 64.99]**	
Total events	7		0				

Heterogeneity: Chi² = 0.01, df = 1 (P = 0.91); I² = 0%
Test for overall effect: Z = 1.97 (P = 0.05)

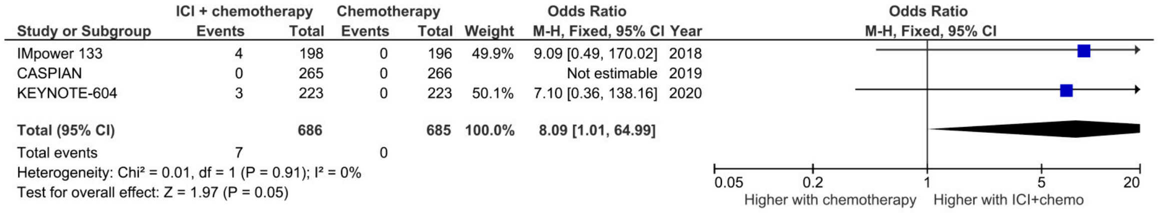

K. Hepatitis, grade 3-4

Study or Subgroup	ICI + chemotherapy Events	ICI + chemotherapy Total	Chemotherapy Events	Chemotherapy Total	Weight	Odds Ratio M-H, Fixed, 95% CI	Year
IMpower 133	3	198	0	196	33.5%	7.04 [0.36, 137.11]	2018
CASPIAN	5	265	0	266	33.1%	11.25 [0.62, 204.54]	2019
KEYNOTE-604	3	223	0	223	33.4%	7.10 [0.36, 138.16]	2020
**Total (95% CI)**		**686**		**685**	**100.0%**	**8.45 [1.55, 46.12]**	
Total events	11		0				

Heterogeneity: Chi² = 0.07, df = 2 (P = 0.97); I² = 0%
Test for overall effect: Z = 2.47 (P = 0.01)

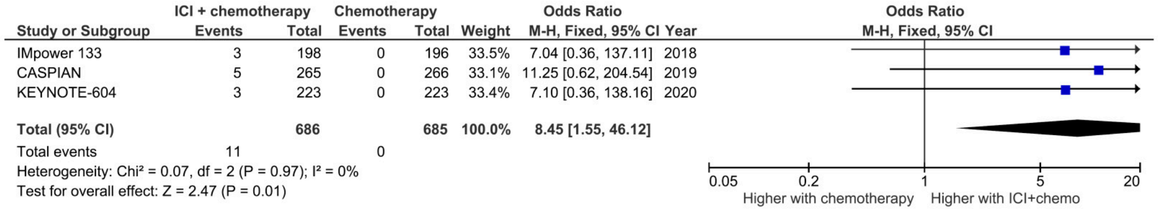

L. Hypothyroidism, all grades

Study or Subgroup	ICI + chemotherapy Events	ICI + chemotherapy Total	Chemotherapy Events	Chemotherapy Total	Weight	Odds Ratio M-H, Fixed, 95% CI	Year
IMpower 133	25	198	1	196	12.2%	28.18 [3.78, 210.15]	2018
CASPIAN	24	265	2	266	25.3%	13.15 [3.07, 56.21]	2019
KEYNOTE-604	23	223	5	223	62.5%	5.01 [1.87, 13.44]	2020
**Total (95% CI)**		**686**		**685**	**100.0%**	**9.90 [4.73, 20.73]**	
Total events	72		8				

Heterogeneity: Chi² = 3.02, df = 2 (P = 0.22); I² = 34%
Test for overall effect: Z = 6.08 (P < 0.00001)

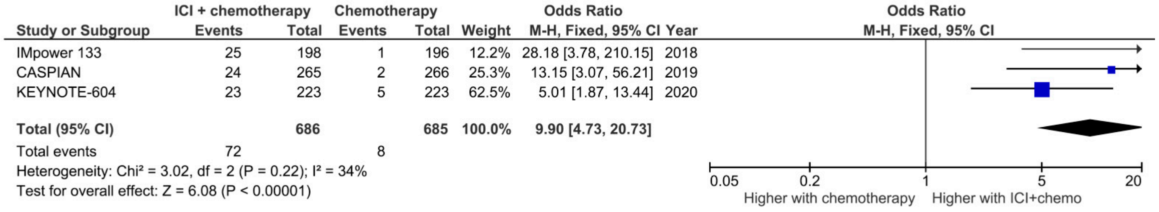

M. Hypothyroidism, grade 3-4

Study or Subgroup	ICI + chemotherapy Events	ICI + chemotherapy Total	Chemotherapy Events	Chemotherapy Total	Weight	Odds Ratio M-H, Fixed, 95% CI	Year
IMpower 133	0	198	0	196		Not estimable	2018
CASPIAN	0	265	0	266		Not estimable	2019
KEYNOTE-604	0	223	0	223		Not estimable	2020
**Total (95% CI)**		**686**		**685**		**Not estimable**	
Total events	0		0				

Heterogeneity: Not applicable
Test for overall effect: Not applicable

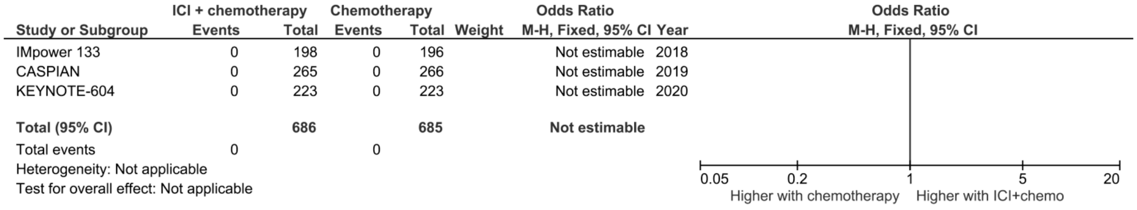

N. Hyperthyroidism, all grades

Study or Subgroup	ICI + chemotherapy Events	ICI + chemotherapy Total	Chemotherapy Events	Chemotherapy Total	Weight	Odds Ratio M-H, Fixed, 95% CI	Year
IMpower 133	11	198	5	196	43.9%	2.25 [0.77, 6.59]	2018
CASPIAN	14	265	0	266	4.4%	30.73 [1.82, 517.85]	2019
KEYNOTE-604	15	223	6	223	51.7%	2.61 [0.99, 6.85]	2020
**Total (95% CI)**		**686**		**685**	**100.0%**	**3.68 [1.89, 7.15]**	
Total events	40		11				

Heterogeneity: Chi² = 3.46, df = 2 (P = 0.18); I² = 42%
Test for overall effect: Z = 3.84 (P = 0.0001)

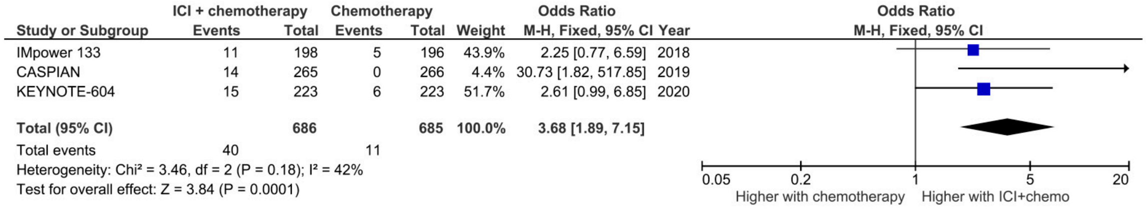

O. Hyperthyroidism, grade 3-4

Study or Subgroup	ICI + chemotherapy Events	ICI + chemotherapy Total	Chemotherapy Events	Chemotherapy Total	Weight	Odds Ratio M-H, Fixed, 95% CI	Year
IMpower 133	0	198	0	196		Not estimable	2018
CASPIAN	0	265	0	266		Not estimable	2019
KEYNOTE-604	1	223	0	223	100.0%	3.01 [0.12, 74.37]	2020
**Total (95% CI)**		**686**		**685**	**100.0%**	**3.01 [0.12, 74.37]**	
Total events	1		0				

Heterogeneity: Not applicable
Test for overall effect: Z = 0.67 (P = 0.50)

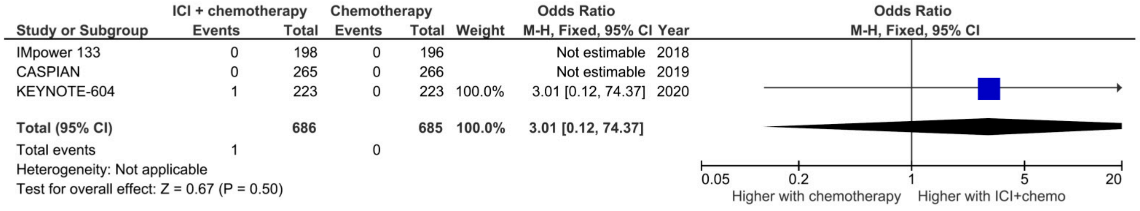

P. Pneumonitis, all grades

	ICI + chemotherapy		Chemotherapy			Odds Ratio	
Study or Subgroup	Events	Total	Events	Total	Weight	M-H, Fixed, 95% CI	Year
IMpower 133	5	198	5	196	42.1%	0.99 [0.28, 3.47]	2018
CASPIAN	7	265	2	266	16.7%	3.58 [0.74, 17.40]	2019
KEYNOTE-604	9	223	5	223	41.2%	1.83 [0.60, 5.56]	2020
**Total (95% CI)**		**686**		**685**	**100.0%**	**1.77 [0.86, 3.63]**	
Total events	21		12				

Heterogeneity: Chi² = 1.59, df = 2 (P = 0.45); I² = 0%
Test for overall effect: Z = 1.56 (P = 0.12)

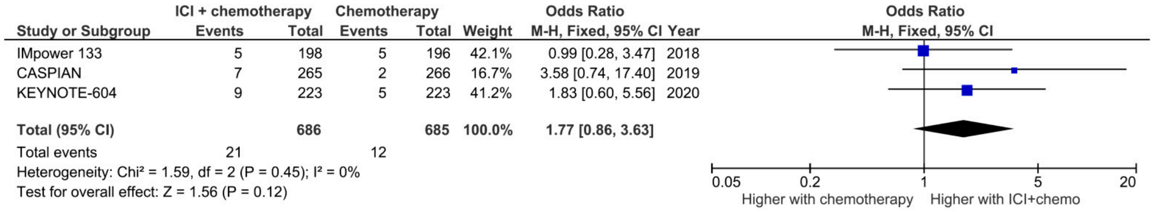

Q. Pneumonitis, grade 3-4

	ICI + chemotherapy		Chemotherapy			Odds Ratio	
Study or Subgroup	Events	Total	Events	Total	Weight	M-H, Fixed, 95% CI	Year
IMpower 133	1	198	2	196	57.4%	0.49 [0.04, 5.47]	2018
CASPIAN	2	265	1	266	28.4%	2.02 [0.18, 22.36]	2019
KEYNOTE-604	3	223	0	223	14.1%	7.10 [0.36, 138.16]	2020
**Total (95% CI)**		**686**		**685**	**100.0%**	**1.86 [0.51, 6.83]**	
Total events	6		3				

Heterogeneity: Chi² = 1.95, df = 2 (P = 0.38); I² = 0%
Test for overall effect: Z = 0.93 (P = 0.35)

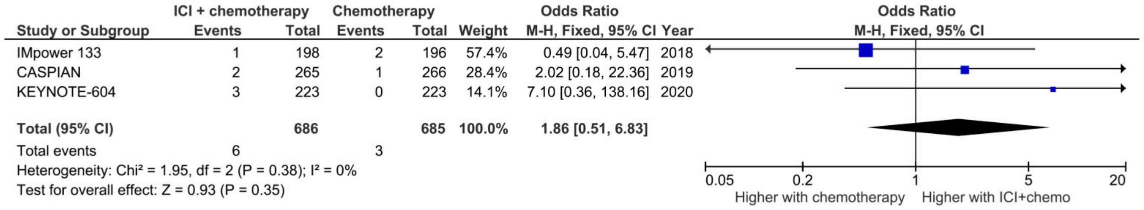

R. Colitis, all grades

	ICI + chemotherapy		Chemotherapy			Odds Ratio	
Study or Subgroup	Events	Total	Events	Total	Weight	M-H, Fixed, 95% CI	Year
IMpower 133	3	198	0	196	14.3%	7.04 [0.36, 137.11]	2018
CASPIAN	4	265	1	266	28.5%	4.06 [0.45, 36.58]	2019
KEYNOTE-604	3	223	2	223	57.2%	1.51 [0.25, 9.11]	2020
**Total (95% CI)**		**686**		**685**	**100.0%**	**3.03 [0.90, 10.19]**	
Total events	10		3				

Heterogeneity: Chi² = 0.96, df = 2 (P = 0.62); I² = 0%
Test for overall effect: Z = 1.79 (P = 0.07)

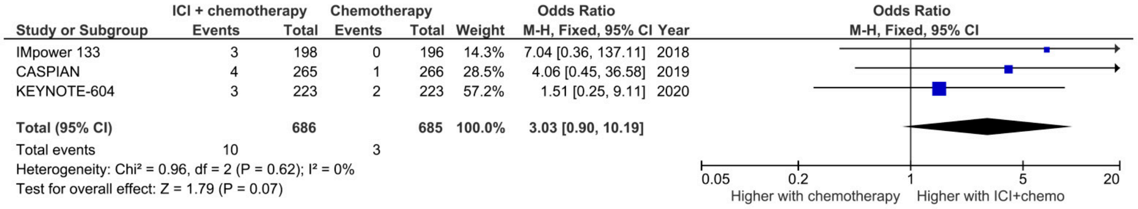

S. Colitis, grade 3-4

	ICI + chemotherapy		Chemotherapy			Odds Ratio	
Study or Subgroup	Events	Total	Events	Total	Weight	M-H, Fixed, 95% CI	Year
IMpower 133	2	198	0	196	16.6%	5.00 [0.24, 104.82]	2018
CASPIAN	1	265	0	266	16.6%	3.02 [0.12, 74.54]	2019
KEYNOTE-604	1	223	2	223	66.7%	0.50 [0.04, 5.53]	2020
**Total (95% CI)**		**686**		**685**	**100.0%**	**1.67 [0.40, 7.00]**	
Total events	4		2				

Heterogeneity: Chi² = 1.60, df = 2 (P = 0.45); I² = 0%
Test for overall effect: Z = 0.70 (P = 0.49)

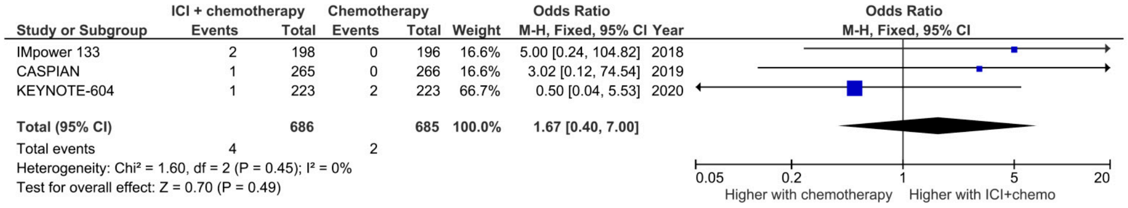

T. Adrenal insufficiency, all grades

	ICI + chemotherapy		Chemotherapy			Odds Ratio	
Study or Subgroup	Events	Total	Events	Total	Weight	M-H, Fixed, 95% CI	Year
IMpower 133	0	198	3	196	70.2%	0.14 [0.01, 2.71]	2018
CASPIAN	1	265	0	266	9.9%	3.02 [0.12, 74.54]	2019
KEYNOTE-604	2	223	1	223	19.8%	2.01 [0.18, 22.32]	2020
**Total (95% CI)**		**686**		**685**	**100.0%**	**0.80 [0.21, 2.97]**	
Total events	3		4				

Heterogeneity: Chi² = 2.56, df = 2 (P = 0.28); I² = 22%
Test for overall effect: Z = 0.34 (P = 0.73)

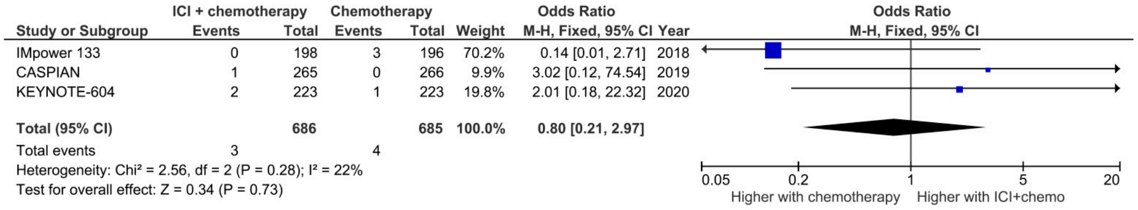

U. Adrenal insufficiency, grade 3-4

	ICI + chemotherapy		Chemotherapy			Odds Ratio	
Study or Subgroup	Events	Total	Events	Total	Weight	M-H, Fixed, 95% CI	Year
IMpower 133	0	198	0	196		Not estimable	2018
CASPIAN	0	265	0	266		Not estimable	2019
KEYNOTE-604	2	223	0	223	100.0%	5.05 [0.24, 105.69]	2020
**Total (95% CI)**		**686**		**685**	**100.0%**	**5.05 [0.24, 105.69]**	
Total events	2		0				

Heterogeneity: Not applicable
Test for overall effect: Z = 1.04 (P = 0.30)

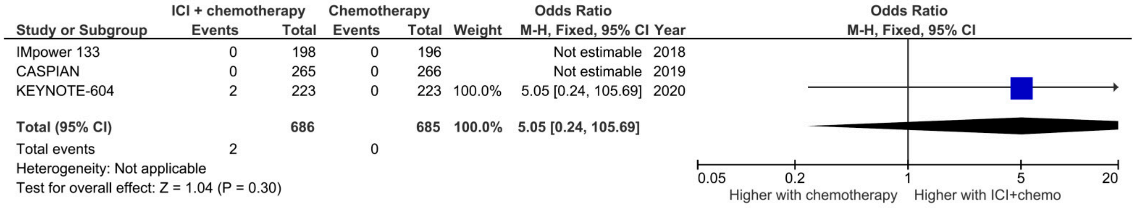

V. Type I diabetes mellitus, all grades

Study or Subgroup	ICI + chemotherapy Events	Total	Chemotherapy Events	Total	Weight	Odds Ratio M-H, Fixed, 95% CI	Year
IMpower 133	1	198	0	196	33.6%	2.98 [0.12, 73.72]	2018
CASPIAN	4	265	0	266	33.0%	9.17 [0.49, 171.21]	2019
KEYNOTE-604	1	223	0	223	33.4%	3.01 [0.12, 74.37]	2020
**Total (95% CI)**		**686**		**685**	**100.0%**	**5.04 [0.87, 29.13]**	
Total events	6		0				

Heterogeneity: Chi² = 0.36, df = 2 (P = 0.83); I² = 0%
Test for overall effect: Z = 1.81 (P = 0.07)

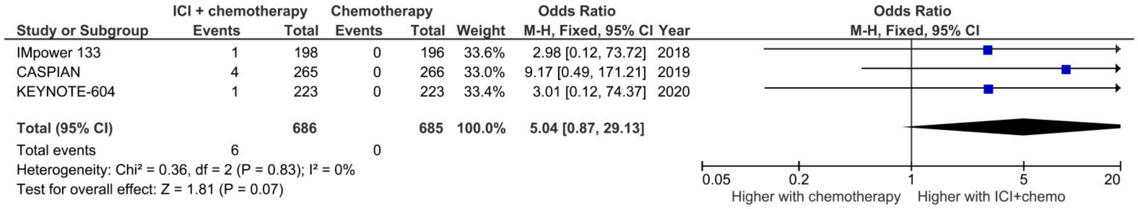

W. Type I diabetes mellitus, grade 3-4

Study or Subgroup	ICI + chemotherapy Events	Total	Chemotherapy Events	Total	Weight	Odds Ratio M-H, Fixed, 95% CI	Year
IMpower 133	0	198	0	196		Not estimable	2018
CASPIAN	4	265	0	266	49.7%	9.17 [0.49, 171.21]	2019
KEYNOTE-604	1	223	0	223	50.3%	3.01 [0.12, 74.37]	2020
**Total (95% CI)**		**686**		**685**	**100.0%**	**6.07 [0.73, 50.57]**	
Total events	5		0				

Heterogeneity: Chi² = 0.26, df = 1 (P = 0.61); I² = 0%
Test for overall effect: Z = 1.67 (P = 0.10)

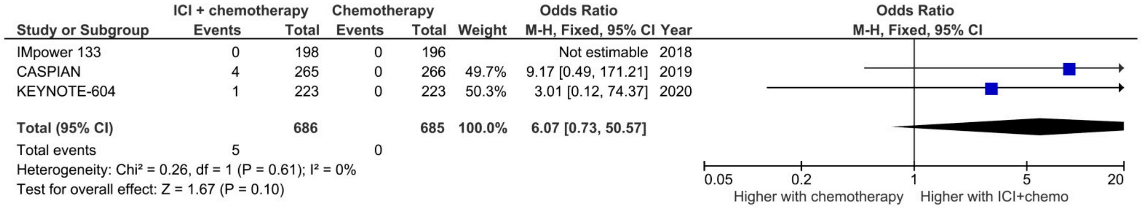

Figure S4. Adverse events recorded in the four trials included in the meta-analysis, in the safety populations. M-H: Mantel-Haenszel; 95% CI: 95% confidence interval.

References

1. Moher D, Liberati A, Tetzlaff J, Altman DG, The PRISMA Group. Preferred Reporting Items for Systematic Reviews and Meta-Analyses: The PRISMA Statement. *PLoS Med*. **2009**, *6(7)*, e1000097, doi:10.1371/journal.pmed1000097.
